# Development and Validation of an Algorithm to Identify Prenatal Care in Administrative Data: Predictive Validity for Adverse Birth Outcomes

**DOI:** 10.1111/1475-6773.70063

**Published:** 2025-10-28

**Authors:** Songyuan Deng, Greg Barabell, Kevin J. Bennett

**Affiliations:** ^1^ South Carolina Center for Rural and Primary Healthcare University of South Carolina School of Medicine Columbia South Carolina USA; ^2^ Clear Bell Solutions Charleston South Carolina USA

**Keywords:** adverse birth outcomes, claims‐based algorithm, hierarchical assignment algorithm, prenatal care continuity, provider attributes

## Abstract

**Objective:**

To develop and validate a hierarchical algorithm for assigning prenatal care (PNC) encounters using claims data while ensuring continuity of care.

**Study Setting and Design:**

We conducted a retrospective cohort study among South Carolina Medicaid beneficiaries. Using a six‐step hierarchical algorithm—incorporating specialty designations, diagnostic/procedure codes, and adjustments for inpatient stays and supplemental visits—we assigned PNC encounters and identified predominant PNC providers. To assess predictive validity, we examined associations between predominant provider status and adverse birth outcomes (obtained from linked birth certificates and claims data) using logit‐binomial generalized estimating equations with robust standard errors, and we compared models' performance using both model fit statistics and 10‐fold cross‐validation.

**Data Sources and Analytic Sample:**

We used South Carolina Medicaid data on live‐birth pregnancies from 2016 to 2021. We followed participants from conception until delivery.

**Principal Findings:**

Initial screening identified 302 package/bundle payment claims, leading to the exclusion of 299 pregnancies (0.3%) from further analysis. The final analytic dataset contained 1,072,615 confirmed PNC encounters for 90,581 (97%) pregnancies. This study identified predominant providers for 87,573 pregnancies (98% of cases with at least two PNC encounters). The analysis of predictive validity revealed significant protective associations for two outcomes when comparing pregnancies with versus without predominant providers: preterm birth (adjusted RR: 0.68, 95% CI: 0.59–0.77) and low‐birth‐weight (adjusted RR: 0.68, 95% CI: 0.57–0.80).

**Conclusions:**

This study developed and validated a claims‐based algorithm to identify PNC utilization in South Carolina Medicaid data. Predictive validity tests revealed that predominant provider status was associated with reduced adverse birth outcomes, suggesting care continuity may improve perinatal health. Future research could apply this algorithm to examine causal relationships between predominant provider status and specific outcomes (e.g., preterm birth, low birth weight), while accounting for institutional and socioeconomic confounders. These findings offer a foundation for optimizing PNC delivery through continuity‐focused interventions.

**Summary**

- What is known on this topic
  ○ Continuity of prenatal care is essential due to healthcare complexity, progressive medical needs, and increasing need for coordination among multiple specialties.
  ○ A recently developed algorithm specifically designed for prenatal care integrated continuity of prenatal care to identify the predominant prenatal care provider.
  ○ Although many claim‐based algorithms can be used to assign prenatal care visit, no algorithm is designed to identify predominant provider based on continuity of prenatal care.
- What this study adds
  ○ We developed a six‐step hierarchical claims‐based algorithm to assign prenatal care encounters, incorporating specific criteria to assess continuity of prenatal care.
  ○ Using the prenatal care information generated by our new algorithm, we applied an existing predominant provider algorithm and successfully identified predominant providers for 94% of included pregnancies.
  ○ Controlling for patient demographics, medical conditions, and prenatal care utilization patterns, we confirmed that predominant provider status significantly predicts adverse birth outcomes.

## 1 Introduction

Prenatal continuity of care (COC) [1] is essential due to healthcare complexity, progressive medical needs, and an increasing need for coordination among multiple specialties. When pregnancy risks are elevated, the probability of a pregnant individual being referred to a specialist increases [2]. In fact, referrals among general pregnant individuals in the US were common [3]. Consequently, the adoption of COC in prenatal care (PNC) is increasing. Better continuity of primary care in preconception was associated with higher subsequent PNC frequency [4]. Additionally, a midwife‐led care model, which ensures continuity by assigning a dedicated midwife to coordinate all care, communications and referrals for pregnant individuals, was associated with improved birth outcomes [5, 6].

Although COC has been widely studied, its basis on the patient's utilization limits its applicability in administrative applications. For instance, if two providers share a significant overlap of patients, their patients' COC measures may appear similar, failing to distinguish between the providers' actual impacts. To address this limitation, the predominant provider concept [7, 8] has been proposed as a provider‐oriented measure of continuity. This approach identifies which provider delivers the predominant number of services to a patient during a specific period, thereby revealing which provider most significantly influences the patient's healthcare utilization and outcomes. Such a provider‐focused COC measure could yield a more meaningful distinction between providers who might otherwise appear similar under patient‐centered continuity measures.

A recently developed algorithm [9] specifically designed for PNC integrated both the sequence and frequency of PNC visits to identify the predominant PNC provider (PPP). This method utilizes PNC frequency to construct density (share of visits to the most‐frequent provider) and dispersion (care concentration adjusted for provider count and total visits) indices during the identification process. The algorithm successfully identified PPP for over 90% of pregnancies, classifying them into four distinct and exclusive subgroups: exclusive providers who deliver all PNC services, major providers who provide more than half of PNC services, plural providers who uniquely deliver the most PNC services (though not exceeding half of total services), and multiple providers who provide both the most PNC services and either the first or last PNC service (Figure S1).

One of the PPP status implications is to detect any association between the COC and birth outcomes, driven by Donabedian's quality framework [10]. Previous publications have proven that patient‐focused COC was associated with birth outcomes, like preterm birth [5, 6]. It is also expected that the provider‐focused COC, measured by the PPP status, should be associated with birth outcomes.

However, identifying the PPP requires detailed and accurate service and provider information. While algorithms leveraging healthcare coding systems in claims data are a forward‐thinking approach, applications of Current Procedural Terminology (CPT) and the International Classification of Diseases (ICD) require specialized training for accuracy. Previous studies have attempted to identify PNC in claims data [11, 12] by including all pregnancy‐related codes, but this method may either under‐ or overestimate PNC frequency or contents, while also potentially misaligning PNC services dates with actual care delivery—all of which could introduce bias into results. For instance, package/bundle payment codes should be excluded from PNC identification because they aggregate multiple service encounters under a single code which may mask the number of providers performing these services. Including these codes would artificially underestimate the PNC frequency and misdate the service delivered. Applying these existing PNC algorithms would lead to misclassification of a provider into an inaccurate PPP status. It is necessary to develop alternatives which align with the PPP status in measuring COC.

This study proposes a novel algorithm to identify PNC using South Carolina Medicaid claims data. The Medicaid program covered 47.4% of live births in South Carolina [13] in 2022 and accounted for 74% of pregnancy‐related deaths [14] during 2018–2020, highlighting the critical importance of this population. This study has three aims: (1) to develop an alternative PNC algorithm for assigning PNC encounters, (2) to assign PPP by applying the proposed PNC algorithm and a previously published PPP algorithm together, and (3) to assess the predictive validity of the proposed PNC algorithm by examining adjusted associations between having a PPP and adverse birth outcomes such as medically unnecessary Cesarean, preterm delivery, and low birth weight.

## 2 Materials and Methods

### 2.1 Data

This study utilized deidentified Medicaid claims data provided by the South Carolina Revenue and Fiscal Affairs (SCRFA) Office. These data include all associated medical claims for enrollees who had a live birth between 2016 and 2021. Available birth certificates were linked to study data by the SCRFA using the beneficiaries' personal identifiers (removed post‐linkage). Birth weights in the linked birth certificates were used as one of the adverse birth outcomes. The study received exemption from the Institutional Review Board at the author's institute, as it involved secondary analysis of de‐identified administrative data.

### 2.2 Sample

A total of 141,464 pregnancies with the conception occurring after October 2015 were identified from claims data, driven by the availability of gestational information in the 10th edition of ICD. Delivery date was confirmed with clinical coding systems. Gestational information was then used to estimate the date of conception (DOC). The method to identify pregnancy episodes was guided by a recently published algorithm [15]. Continuous coverage from the DOC through delivery was applied to exclude pregnancies that may have missed visit information due to coverage gaps or Medicaid as a secondary payer. This approach results in a total of 93,135 pregnancies, representing 77,144 pregnant participants. Detailed sample attrition can be found in Table S1. The retrospective cohort study design was applied to the current study.

### 2.3 Assigning Prenatal Care

Three fields of information were used simultaneously to confirm a PNC encounter: (1) a provider (aggregated using physical address) with a professional specialty capable of providing PNC, (2) a diagnosis related to pregnancy or pregnancy complications, and (3) a procedure related to obstetric care. Depending on the research purpose and the source of claims data, the specialties included may vary. In this study, in addition to obstetrics/gynecology (OBGYN) specialists, primary care and emergency physicians, physician assistants, midwives, nurse practitioners, and federally or state funded medical clinics (Federally Qualified Health Centers, Rural Health Clinics, and Department of Health and Environmental Control, referred to as FQHCs, RHCs, and DHEC, respectively) are included in identifying PNC. Details of the specialties included can be found in Table S1.

For diagnosis, only the primary diagnosis was used to confirm that the main reason for an encounter was PNC, using three categories of ICD codes related to pregnancy or pregnancy complications: (1) Chapter Z for confirmation of pregnancy test, pregnancy state, supervision of normal pregnancy, antenatal screening, and weeks of gestation; (2) Chapter N for absent or irregular menstruation (first PNC encounter only); and (3) Chapter O for conditions related to pregnancy and childbirth. Details of the ICD codes included can be found in Table S1.

Six families of CPT codes can be used to identify PNC procedures. These include Evaluation and Management (E/M), Maternity Care and Delivery under Surgery (MCD), Behavioral Health and/or Substance Abuse Treatment Services from CPT Category II (BHSATS), Patient Management from the Healthcare Common Procedure Coding System (HCPCS) level II (PM), Pathology and Laboratory (P/L) and Radiology. Details of the included CPT/HCPCS codes can be found in Table S1.

This algorithm utilizes an encounter‐based methodology that classifies six CPT code families according to their functional role: MCD codes serve as exclusion criteria for pregnancy, E/M and PM codes constitute essential encounter indicators, BHSATS codes are treated as optional components, while P/L and radiology codes provide supplemental data. This hierarchical approach ensures the algorithm prioritizes clinically meaningful encounters while systematically accounting for exclusions, optional elements, and ancillary services.

A six‐step approach was implemented (Table S1). First, we excluded pregnancies containing MCD codes. Second, we established the first PNC encounter using all codes included. Steps three and four respectively confirmed subsequent PNC encounters (E/M/BHSATS/PM codes) and supplemental visits (P/L/radiology codes). Through steps two to four, we applied priority rules: OBGYN specialists took precedence over midwives, and inpatient settings were prioritized over emergency departments.

The fifth step is to remove redundant visits at the inpatient setting. PNC requires ongoing clinical supervision of fetal and maternal health, which cannot be interrupted by prolonged hospitalization. This study adjusted PNC encounters using a 13‐day threshold (adopted from a previous study [16] as the maximum average prenatal hospitalization duration across conditions). Stays under 13 days were counted as one encounter; stays exceeding this threshold were divided into 13‐day intervals, with each interval counted as one encounter. This approach balances granularity with the practical need to quantify care continuity during hospitalization.

The final step involved defining intervals for supplemental visits (P/L/radiology codes) to minimize overcounting of PNC. Because overlapping codes for these services and a preceding PNC encounter could inflate counts, a time‐based separation threshold is required. PNC phases are outlined by the American College of Obstetricians and Gynecologists (ACOG): monthly visits before 28 weeks, biweekly until 36 weeks, and weekly thereafter. Accordingly, intervals were set to 14 days prior to 36 weeks, and 7 days thereafter, ensuring consistency with ACOG guidelines while mitigating duplication.

### 2.4 Application and Statistical Analysis

For each algorithmic step, we will report both the number of identified PNC encounters and the count of pregnancies with any PNC detection. PNC encounters were aggregated to the pregnancy level. Key metrics will be reported for gestational age in days at first PNC encounters and total PNC frequency (with mean ± SD). These measures will quantify population‐level PNC engagement patterns while assessing the algorithm's performance across the pregnancy cohort.

Using the assigned PNC encounter data, this study applied a developed PPP algorithm [9] to determine the PPP for each pregnancy. Predominant providers were classified based on three key dimensions of care continuity: PNC density, dispersion, and temporal sequence. This classification system categorized pregnancies into five mutually exclusive groups: those with either an exclusive (100% of visits), major (> 50% of visits), plural (uniquely largest share but ≤ 50% of visits), multi‐plural predominant provider (largest share but ≤ 50% of visits, and the first or last visit), or without a predominant provider (Table S1).

To examine the predictive validity of assigned PNC encounters by the proposed algorithm, this study assessed the association of assigned predominant providers with three adverse birth outcomes: medically unnecessary cesarean delivery (defined per ACOG guidelines [17] as non‐indicated procedures), preterm birth (live birth before 37 completed gestational weeks), and low birth weight (< 2500 g, verified through linked birth certificates). All outcomes were coded as binary variables (1 = present, 0 = absent). This analysis tested whether provider patterns identified by our algorithm demonstrated clinically meaningful predictive capacity for these adverse birth outcomes.

Adverse birth outcomes were examined at the pregnancy level, comparing outcomes between pregnancies with versus without a PPP using a logit‐binomial generalized estimating equation (GEE) model with robust standard errors. The first regression included a binary variable to indicate any predominant provider or not (full‐binary), and the second regression included a categorical measure for all provider subgroups (full‐category). The adjusted model controlled for maternal age at delivery, race, rurality of resident zip code (classified with 2020 Rural–Urban Commuting Area codes at the ZCTA level, categorizing codes 1.0–3.0, 4.1, 5.1, 7.1, 8.1, and 10.1 as urban and all others as rural), estimated gestational week of PNC initiation, estimated PNC frequency, group consultation, severe maternal morbidity (yes or no) [18], maternal comorbidity score [19], history of adverse pregnancy experiences (ICD‐10, O090‐O092, O09A, O0982), and current pregnancy risk factors (ICD‐10, O093‐O097, O0981, O0989, O099). Risk ratios (RR) with 95% confidence intervals (CI) were reported. We compared the two full models against a reduced model (which excluded the predominant provider measure) using the quasi‐likelihood information criterion (QIC) and 10‐fold cross‐validated area under the receiver operating characteristic curve (AUC). QIC evaluates model fit and complexity for robust parameter estimation, and the AUC assesses predictive accuracy and generalizability. Statistical analyses were conducted using SAS software version 9.4 (SAS Institute Inc., Cary, NC) with a significance level set at 95%.

## 3 Results

Characteristics of study participants can be found in Table S1. Table 1 presents the stepwise confirmation of PNC visits using the proposed algorithm. Initial screening identified 302 MCD claims, leading to the exclusion of 299 pregnancies (0.3%) from further analysis. The raw 1,228,594 PNC encounters included 95,663 first visits (step 2), 942,209 PNC encounters (step 3), and 190,722 supplemental visits (step 4). Application of the sequential adjustment criteria refined these counts as follows: (1) specialty and setting prioritization eliminated 11,900 duplicate records through steps 2–4, (2) inpatient interval exclusion removed an additional 1069 visits in step 5 (primarily from PNC encounters), and (3) supplemental visit interval filtering excluded 143,010 records in step 6. The final analytic dataset contained 1,072,615 confirmed PNC encounters.

**TABLE 1:** Prenatal care visits confirmed with various codes and adjustments.

Step	Step 1	Pregnancy	All visits	Step 2	Step 3	Step 4
Exclusion	302	299	N.A.^a^	N.A.	N.A.	N.A.
Raw code	N.A.	90,581	1,228,594	95,663	942,209	190,722
Adjustment 1 for specialty and setting	N.A.	90,581	1,216,694	90,581	935,573	190,540
Step 5, adjustment 2 for inpatient interval	N.A.	90,581	1,215,625	90,581	934,509	190,535
Step 6, Adjustment 3 for supplemental intervals	N.A.	90,581	1,072,615	90,581	934,509	47,525

*Note:* The total sample size was 93,135. Step 1: to exclude pregnancies with any package/bundle codes. Step 2: The first visit was identified with a positive pregnancy test, prenatal care (PNC) encounters, and supplemental PNC encounters. Step 3: a PNC encounter was confirmed with Evaluation/Management and PM (Patient Management from the Healthcare Common Procedure Coding System Level II) codes, with optional BHSATS (Behavioral Health and/or Substance Abuse Treatment Services from the Current Procedural Terminology Category II) codes. Step 4: supplemental PNC was confirmed with Pathology/Laboratory and radiology codes. Adjustment 1: this adjustment was implemented through Steps 2–4. Specialty priority is Obstetrics and Gynecology, Primary Care Physician, Physician Assistant, Midwife, Nurse Practitioner, and Organizations (Federally Qualified Health Centers [FQHC]/Rural Health Clinics [RHC]/Clinics of Department of Health and Environmental Control [DHEC]). Setting priority is Office, Clinic, Inpatient, Emergency Room, and Telehealth. Step 5: Adjustment 2, this study adjusted PNC encounters using a 13‐day threshold (adopted from a previous study as the maximum average prenatal hospitalization duration across conditions). Stays under 13 days were counted as one visit; stays exceeding this threshold were divided into 13‐day intervals, with each interval counted as one visit. Step 6: Adjustment 3, intervals were set to 14 days prior to 36 weeks, and 7 days thereafter.

^a^ Not available.

Table 2 presents pregnancy‐level PNC metrics derived from the proposed algorithm. After excluding those with MCD codes, we identified 2255 (2.4%) pregnancies with no assigned PNC. The remaining 90,581 pregnancies accounted for 1,072,615 PNC encounters. Key utilization metrics revealed: (1) the mean gestational age at PNC initiation was 71 days (SD = 48) and (2) the mean number of PNC encounters per pregnancy was 12 (SD = 5), demonstrating substantial variation in both care initiation timing and service utilization intensity across the cohort.

**TABLE 2:** Prenatal care metrics identified with the proposed algorithm, at the pregnancy level.

Step/metric	Pregnancies (%)	PNC^a^ frequency	
		Mean	SD
W/ continuous coverage	93,135 (100.0)	N.A.^b^	N.A.
W/o package/bundle codes	92,836 (99.7)	N.A.	N.A.
Had any PNC	90,581 (97.3)	11.8	5.0
Gestational days at the first PNC		71.3	47.9
W/ a predominant provider, *n* (%)	87,573 (94.0)	12.2	4.7
With an exclusive provider	13,181 (14.2)	9.2	4.2
With a major provider	56,882 (61.0)	12.7	4.4
With a plural provider	13,891 (14.9)	13.2	5.2
With a multi‐plural provider	3619 (3.9)	11.1	4.7
W/o a predominant provider, *n* (%)	5562 (6.0)	1.5	3.0
W/ package/bundle codes	299 (0.3)	N.A.	N.A.
No PNC at all	2255 (2.4)	0.0	0.0
W/ only one PNC	1551 (1.7)	1.0	0.0
Others	1457 (1.6)	4.4	4.4

^a^ PNC, prenatal care.

^b^ N.A, not available.

Using the PNC information from this algorithm, this study then applied the PPP algorithm to identify predominant providers for 87,573 pregnancies (94% of eligible cases). Among these, provider distribution followed four distinct patterns: exclusive providers accounted for 13,181 pregnancies (14%), major providers for 56,882 (61%), plural providers for 13,891 (15%), and multi‐plural providers for 1619 (4%). After excluding 299 pregnancies (0.3%) with MCD claims and 3806 pregnancies (4%) with fewer than two PNC encounters, only 1457 pregnancies (< 2%) lacked an identifiable predominant provider. The PNC frequency was not linearly associated with the predominant provider levels (Table 2).

**FIGURE 1:**
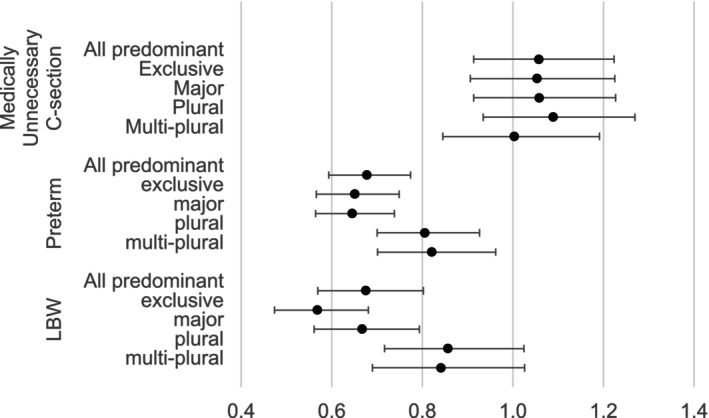
Provider‐type differences in adverse birth risk: adjusted risk ratios with 95% confidence intervals. The analysis was conducted among pregnancies with at least two prenatal care visits, with a sample size of 89,030. The sample size of low birth weight was 75,307, limited to those with available birth weight recorded in the birth certificate. Pregnancies were classified into five groups: with an exclusive (provides all prenatal care), a major (provides > 50% prenatal care), a plural (uniquely provides the most prenatal care among all providers), or a multi‐plural predominant provider (provides the most prenatal care among all providers, and provides the first or the last care), and without a predominant provider (the reference group). All predominant includes all subgroups of predominant providers: exclusive, major, plural, and multi‐plural predominant provider. The analysis was conducted with a logit‐binomial generalized estimating equation (GEE) model with robust standard errors. The first regression included a binary variable to indicate any predominant provider or not, and the second regression included a categorical measure for all provider subgroups. The adjusted model controlled for maternal age at delivery, race, rurality of resident zip code, severe maternal morbidity, maternal comorbidity score, history of adverse pregnancy experiences, current pregnancy risk factors, the gestational week of the first prenatal care encounter, the total number of prenatal care encounters and group consultation.

Figure 1 displays adjusted RRs with 95% CI for adverse birth outcomes by predominant provider status, revealing significant protective associations for two outcomes when comparing pregnancies with versus without predominant providers. For preterm births, pregnancies with a predominant provider showed an adjusted RR of 0.68 (95% CI: 0.59–0.77) compared to those without a predominant provider. Similarly, for LBW, the adjusted RR was 0.68 (95% CI: 0.57–0.80) for predominant providers. No statistically significant differences were observed in rates of medically unnecessary cesarean deliveries across provider groups.

Table 3 summarizes the model comparisons between the reduced model and the two full models. Both QIC and AUC metrics demonstrated that the reduced model was most efficient for predicting medically unnecessary cesarean sections, while the full‐categorical model showed the best performance for preterm births and low birth weight among the three models. For medically unnecessary cesarean sections, the reduced model had the lowest QIC values (76,416 for reduced vs. 76,418 for full‐binary and 76,421 for full‐categorical), and neither full model showed significant improvement in AUC difference. In contrast, for preterm births, the full‐categorical model achieved the lowest QIC (64,952 for full‐categorical vs. 65,060 for reduced and 65,028 for full‐binary) and demonstrated a statistically significant improvement in AUC (difference = 0.0016, 95% CI: 0.0002–0.0030). Similarly, for low birth weight, the full‐categorical model yielded the lowest QIC (46,863 vs. 46,976 for reduced and 46,958 for full‐binary) and significantly improved AUC (difference = 0.0031, 95% CI: 0.0015–0.0047).

**TABLE 3:** Model performance comparison between reduced and full models.

Model performance	Medically unnecessary C‐section	Preterm	Low birth weight
QIC			
Reduced	76416.5	65060.2	46975.7
Full‐binary	76418.0	65027.9	46957.6
Full‐category	76421.3	64952.0	46862.8
VC—AUC			
Reduced (ref.)	0.62086	0.70615	0.68011
Full‐binary	0.62082	0.70672	0.68065
Diff‐mean	−0.00005	0.00057	0.00054
Lower 95% CL	−0.00016	0.00003	0.000003
Upper 95% CL	0.00006	0.00111	0.00107
Full‐category	0.61285	0.70776	0.68319
Diff‐mean	−0.0001	0.00161	0.00308
Lower 95% CL	−0.0004	0.00024	0.00150
Upper 95% CL	0.00021	0.00298	0.00465

*Note:* Reduced model included all covariates, including maternal age at delivery, race, rurality of resident zip code, severe maternal morbidity, maternal comorbidity score, history of adverse pregnancy experiences, current pregnancy risk factors, the gestational week of the first prenatal care encounter, the total number of prenatal care encounters and group consultation.

Full‐binary model added a binary measure of predominant prenatal care provider into the reduced model. Full‐category model added a categorical measure of predominant prenatal care provider into the reduced model.

## 4 Discussion

This study developed and validated an algorithm for identifying PNC utilization patterns in South Carolina Medicaid claims data through comprehensive analysis of clinical coding systems. This hierarchical methodology identified PNC for 97% of pregnancies. By applying the PPP algorithm to these identified PNC, the study assigned predominant providers for 94% of cases. Predictive validity testing at the pregnancy levels revealed clinically significant patterns: providers serving as predominant providers demonstrated substantially better birth outcomes compared to non‐predominant provider relationships. The model comparisons revealed that for both preterm births and low birth weight, the models incorporating the predominant provider as a categorical measure provided a superior model fit and a statistically significant improvement in predictive accuracy.

A recent review summarized all existing methods for assigning PNC using claims data [20]. The summarized methodologies either included all encounters or were designed to access PNC content. Only the pregnancy or patient perspective was considered in those methodologies. To assign predominant providers—derived from the COC with a provider perspective [9, 21, 22]—this study established a hierarchical algorithm. In strict adherence to clinical coding guidelines [23, 24, 25, 26, 27] this study excluded both MCD codes and pregnancy test codes. To align with ACOG guidelines and the COC framework, this study incorporated specific adjustments for inpatient stays and supplemental visit data. Evidence of the positive association between predominant providers and COC metrics can be found in the supplemental file.

This algorithm evaluates how well claims‐based PNC identification predicts birth weights (linked birth certificates) and preterm births (estimated from claims). The adjusted regression model controlled for patients' demographics, medical conditions, and related PNC services. Despite these adjustments, the RRs for both preterm births and LBW demonstrated a moderate protective effect from the application of the developed algorithm. Future studies could further investigate predictive validity using more advanced study designs or by controlling for socioeconomic factors.

Our analysis identified predominant providers for 94% of pregnancies with assigned PNC. These predominant providers present a focused target for quality improvement initiatives by policymakers, payers, and healthcare systems. Importantly, non‐predominant providers play vital complementary roles—while predominant providers function as care hubs, other providers crucially expand access to underserved populations. Future research should investigate how referral networks between predominant and non‐predominant providers can optimize both care continuity and geographic accessibility, particularly in rural or resource‐limited settings.

These findings highlight the critical need for hierarchical code inclusion in claims‐based PNC studies. Indiscriminate use of clinically relevant codes without rigorous validation may undermine research validity, leading to either under‐ascertainment or false‐positive case identification. However, the applicability of this algorithm is limited to insurance plans that reimburse individual PNC encounters, as it cannot accommodate bundled or packaged service payment structures. The proposed PNC identification algorithm was applied to pregnancies with any global/bundle/package claims. These pregnancies had an average PNC frequency of 6.39 encounters, which was significantly fewer than the average of 11.8 encounters in the included population. PNC information was limited in claims data among pregnancies with these payment types. This result demonstrates that the proposed algorithm cannot be reliably applied to these pregnancies. Furthermore, the proposed PNC algorithm was examined only in South Carolina Medicaid claims; applications in other states' Medicaid or other healthcare systems require additional adjustments in specific codes to align with specific reimbursement policies. Whether the derived PPP status was still consistently associated with adverse birth outcomes also needs to be examined separately.

The predictive validity analysis demonstrated significant associations between predominant provider status and reduced adverse birth outcomes, supporting the algorithm's utility for investigating PNC utilization patterns. These findings suggest that a provider's predominant status in care delivery was associated with birth outcomes. The role of a predominant provider exhibits important dynamic qualities in explaining the adverse birth outcomes. The current study design did not determine temporal precedence in this relationship: we cannot establish whether (1) initial predominant status leads to later quality improvement or (2) pre‐existing high quality enables providers to achieve and maintain predominance. This critical distinction warrants future longitudinal research tracking provider trajectories before and after attaining predominant status, while accounting for organizational and financial factors that may influence these transitions.

A key limitation of this study is its focus on the extensive margins of preterm birth and low birth weight. While this identifies factors associated with the occurrence of these adverse outcomes, it does not capture their severity. We choose the binary outcomes for two reasons. The first one is that these binary definitions are well recognized and applied in many initiatives like the Title V Maternal and Child Health Services Block Grant. The results can be directly applied by those initiatives. The second reason is that low birth weight is measured for each new baby, not for delivery. Our study assigned a presence of low birth weight for a delivery if any baby was weighed less than 2500 g. Consequently, our findings may not fully reflect the nuanced relationship. Future studies may incorporate analyses of intensive margins to provide a more comprehensive understanding.

When combining the PNC algorithm proposed in the current study and the PPP algorithm published previously, the PPP status can be applied in studies for healthcare workforce and utilization and health outcomes. For example, the differences in travel burden for South Carolina rural residents were smaller to the PPP than to the nearest provider, compared to that for urban residents [28]. Regarding determinants, researchers can investigate which patient and provider characteristics are associated with PPP status. For policymakers, this metric is a practical tool for surveillance and improving access to PNC. For example, it was found that 6% of pregnancies without a PPP had extremely low PNC utilization. It can also be used to evaluate cost‐effectiveness; 15% of pregnancies with a plural provider had the highest utilization rates but were linked to only a modest reduction in preterm birth risk and no significant association with low birth weight. Finally, the PPP status can guide evidence‐based interventions—such as deploying PNC navigators to coordinate care and improve continuity, particularly for high‐risk patients—to reduce adverse birth outcomes.

## 5 Conclusions

This study developed and validated a claims‐based algorithm to identify PNC utilization in South Carolina Medicaid data. Predictive validity tests revealed that predominant provider status was associated with reduced adverse birth outcomes, suggesting care continuity may improve perinatal health. Future research could apply this algorithm to examine causal relationships between predominant provider status and specific outcomes (e.g., preterm birth, low birth weight), while accounting for institutional and socioeconomic confounders. These findings offer a foundation for optimizing PNC delivery through continuity‐focused interventions.

## Conflicts of Interest

The authors declare no conflicts of interest.

## Supporting information


**Data S1:** hesr70063‐sup‐0001‐Supinfo.docx.

## Data Availability

The South Carolina Medicaid data that support the findings of this study are available from the South Carolina Revenue and Fiscal Affairs Office, but restrictions apply to the availability of these data, which were used under license for the current study, and so are not publicly available. Software programs are available upon request to the corresponding author through email: songyuan@email.sc.edu.
