# Supplementary material for: Development and Validation of an Algorithm to Identify Prenatal Care in Administrative Data: Predictive Validity for Adverse Birth Outcomes
Source: Health Serv Res. 2025 Oct 28;61(1):e70063. doi: 10.1111/1475-6773.70063 (PMC12857457; doi:10.1111/1475-6773.70063)
Supplement: Supplementary file 1 — Data S1: hesr70063‐sup‐0001‐Supinfo.docx. [file HESR-61-0-s001.docx]

Supplemental Figure 1. Predominant provider status in different pregnancy scenarios

Exclusive provider: A

A

A

A

A

A

A

A

A

A

A

A

A

A

A

A

B

B

B

B

A

Major provider: A

A

A

A

A

B

B

B

C

C

C

Plural provider: A

A

B

B

B

C

C

C

D

A

A

Multiple-plural provider: A

A

B

C

D

E

F

G

H

I

J

Over-dispersed:

A

B

B

B

B

C

C

C

C

A

No predominant provider:

Note: Exclusive providers deliver all prenatal care (PNC) services. Major providers provides more than half of PNC services, Plural providers uniquely deliver the most PNC services (though not exceeding half of total services). Multiple providers provide both the most PNC services and either the first or last PNC service.

Supplemental Table 1. Sample attrition flow.

| Number of pregnancy episode | Exclusion criteria | Excluded number of episodes |
| --- | --- | --- |
| Day of Conception after 2015 October: 141,464 |  |  |
|  | Medicaid is not the primary payor | 17,157 (12.1%) |
|  | Without continuous coverage | 31,172 (22.0%) |
| With continuous coverage: 93,135 |  |  |

Note: Two key limitations may affect claims completeness: (1) patients with coverage gaps may not submit claims during uninsured periods, and (2) even continuously enrolled patients may not bill Medicaid for services when it serves as a secondary payer.

Supplemental Table 2

Codes for identification of prenatal care in South Carolina Medicaid beneficiaries

| Field / Coding system | Group | Code | Notes |
| --- | --- | --- | --- |
| Provider / Provider Specialty | Obstetrics & Gynecology | 16, 26, 27, OB | Must included |
|  | Primary care physician | 12, 14, 19, 78 |  |
|  | Emergency Medicine | 10 |  |
|  | Physician Assistant | PA |  |
|  | Midwife | 06 |  |
|  | Nurse practitioner | 86 |  |
|  | FQHC | 50 |  |
|  | RHC | 97 |  |
|  | DHEC | 51 |  |
| Primary diagnosis / ICD | Absent, irregular menstruation | N911, N912, N926 | Only for the first prenatal care |
|  | Health status | Z3200, Z3201, Z331 | Only for the first prenatal care, including secondary diagnoses |
|  |  | Z34, Z36, Z3A | Must included |
|  | High-risk pregnancy, pregnant complications | O09, O1, O2, O3, O4, O9811, O9821-O9891 (6^th^ digit was 1,2,3,9), O9901, O9911, O9921 (6^th^ digit was 0-3), O9928 (6^th^ digit was 0-3), O9931- O9935(6^th^ digit was 0-3), O9941, O9951, O9961, O9971, O99810, O99820, O99830, O9984 (6^th^ digit was 0-3), O99891 | Must included |
| Procedures / CPT & HCPCS | Package/ bundle as MCD codes | 59400, 59425, 59426, 59510, 59610, 59618 | Exclusion criteria |
|  | E/M codes | 99201-99205, 99211-99215, 99221-99223, 99231-99236, 99281-99285, 99341-99345, 99347-99350, 98966-98968, 98970-98972, T1015 | Must included |
|  | PM codes | H1001 |  |
|  | BHSATS codes | 0500F, 0501F, 0502F | Optional |
|  | P/L codes – pregnancy test | 81025, 84702, 84703, 84704 | Not included |
|  | P/L codes – monitoring fetal status | 59000, 59001, 59012, 59015, 59020, 59025, 59500, 59030, 59070, 59072, 59074, 59076, 81420, 81507, 81422, 81508-81522, 84163, 84112, | Included with intervals |
|  | P/L codes – screening for teratogen exposures, infection | 80081, 87389, 86762, 3292F, 80055, 87340, 3513F, 86592, 3512F, 86762, 87270, 87320, 87485-87487, 87490-87492, 87810, 3511F, 87590-87592, 87850, 86480, 86580, 87555-87557, 3510F |  |
|  | P/L codes – screening for teratogen exposures, vaccination | 90389, 90697, 90698, 90714, 90715, 90723, 99281, 90630, 90647, 90648, 90653, 90654, 90655, 90656, 90657, 90658, 90661, 90662, 90666, 90667, 90668, 90673, 90674, 90756, 90682, 90685, 90686, 90687, 90688, 90748, 1030F, 4035F, 4037F, 4274F, 90396, 80055, 80081, 86762, 90632, 90633, 90634, 90636, 4148F, 80074, 86708, 86709, 90371, 90636, 90697, 90723, 90739, 90740, 90743, 90744, 90746, 90747, 90748, 4149F, 86704, 86705, 86706, 86707, 87340, 90620, 90621, 90733, 90734, 90670, 90732, 4040F |  |
|  | P/L codes – screening for teratogen exposures, substance abuse | 80320, 80321, 80322, 99408, 99409, 3016F, 1000F, 99408, 99409, 80346, 80347, 80349, 90350, 80351, 80352, 80353, 80354, 80356, 80357, 80358, 80361, 80362, 80363, 80364, 80365 |  |
|  | Radiology | 76801, 76802, 76805,76810, 76817, 76811, 76812, 76813, 76814, 76815, 76816, 76825, 76826, 76827, 76828, 76818, 76819, 76820, 76821, 76818, 76941, 76945, 76946, 74712, 74713 |  |

Note: E/M codes can be classified into four groups: general services (for different settings and levels of medical decision making), non-Face-to-Face services, medical team conference, and special services. In the identification of prenatal care, only the E/M codes of general services and non-Face-to-Face services are used.

Furthermore, not all general E/M codes from all settings are applicable to prenatal care. Acceptable settings include office or outpatient, hospital inpatient, emergency department, and home services. If medical clinics funded by the federal or states are included, a specific code (T1015) from HCPCS level II may also be included. Although prolonged services are valid codes, they must be used in conjunction with other E/M codes which could lead to unnecessary encounter duplication. Non-Face-to-Face services include telephone services and online medical evaluation. Cautions should be applied here, as the CPT updated codes related to telemedicine in response to the COVID-19 pandemic.

MCD codes would not be used after careful examination. For example, antepartum, delivery and postpartum care can be bundled in different ways to code for healthcare services received by patients. However, routine obstetric care package -- where a single code covers all obstetric care for uncomplicated pregnancies -- cannot be used to identify prenatal care in claims data. Therefore, the percentage of beneficiaries whose claims contain these routine obstetric care packages should be examined before applying CPT codes. Only if that percentage is reasonable can the CPT codes be applied on claims data to identify prenatal care, excluding those participants. Three codes require special attention: code 59425 indicates 4-6 prenatal cares visits, code 59426 indicates 7-14 prenatal care visits, and the modifier 22 with 59426 suggests 15 or more visits. However, this study does not recommend using any MCD code for the identification of prenatal care.

Other recommended codes for individual prenatal care include BHSATS code. However, because CPT category II codes are optional and supplemental, they do not represent actual encounters and care and their availability does not impact reimbursement, not all providers utilize these codes. As a result, while these codes can be included in the identification of prenatal care, they are not considered consistently reliable information sources and should only be used in conjunction with other codes.

PM codes for prenatal care are primarily used by Medicaid state agencies as a methodology to provide enhancement payment for directed initiatives. Unlike BHSATS codes, there would be a much higher likelihood of reporting these HCPCS level II codes as they are associated with a link of service to payment. For instance, code H1001, “Prenatal care, at-risk enhanced service; antepartum management” was used by SC Medicaid during 2016 – 2023 to provide enhanced prenatal and postpartum home visitation services under the Nurse Family Partnership (NFP) model.

Many P/L codes are universal rather than specifically designed for prenatal care. However, during a confirmed pregnancy episode with a live birth, procedures such as pregnancy testing, monitoring fetal status, and screening for potential teratogen exposures (including infections, vaccinations, and substance use) are more likely to be used during a prenatal visit. Medical directions or prescriptions may have been issued on the day of an E/M encounter, but these procedures could be carried out several days or weeks later. Therefore, P/L codes serve as supplemental information to E/M codes in identifying prenatal care.

Among P/L codes, although codes for pregnancy tests are legitimate, they represent the procedure rather than the result. A negative result, even with pregnancy tests, means no start of prenatal care. Positive or unknown results of pregnancy tests may suggest the start of prenatal care. These results can be identified using ICD codes. However, absence of these codes does not mean a pregnancy test was negative.

The final group is radiology. Ultrasound and Magnetic Resonance Imaging (MRI) for fetal evaluation and guided invasive procedures can also be used in prenatal care. Including radiology codes increases the accuracy of identifying the initiation of prenatal care because dating ultrasound is used to estimate the due date and rule out ectopic pregnancy. This ultrasound report can be utilized for setting up a plan for future prenatal care.

Supplemental Table 3. Six steps of assigning prenatal care with claims information.

| Step 1 | To exclude pregnancies with any package/bundle codes; |
| --- | --- |
| Step 2 | To assign the first encounter with positive pregnancy test, PNC encounters, and supplemental PNC encounters, adjusted for specialty priority and setting priority; |
| Step 3 | To assign following PNC encounters with E/M and PM codes, with optional BHSATS codes, adjusted for specialty priority and setting priority; |
| Step 4 | To assign supplemental PNC encounters with P/L and radiology codes, adjusted for specialty priority and setting priority; |
| Step 5 | To adjust inpatient PNC encounters using a 13-day threshold: stays under 13 days were counted as one visit, stays exceeding this threshold were divided into 13-day intervals, with each interval counted as one visit; |
| Step 6 | To adjust supplemental PNC encounters using a 14-day interval prior to 36 gestational weeks, and a 7-day interval thereafter. |

Supplemental Table 4. Classification of pregnancy episodes by predominant providers.

| Pregnancy episodes | Description | Formula |
| --- | --- | --- |
| With a predominant provider |  |  |
| An exclusive provider | Provides all PNC services | M =1 |
| A major provider | Provides >50% PNC services | $\frac{max(n_{i})}{N}$ > 50% |
| A plural provider | Uniquely provides the most PNC services among all PNC providers | $n_{i}= max(n_{i})$,  and count $max\left( n_{i} \right)=1$ |
| One of multiple providers | Provides the most PNC services among all PNC providers, and provides the first or the last PNC service | $n_{i}= max(n_{i})$,  count $max\left( n_{i} \right)>1$,  and $P{NC}_{1st}=1 or P{NC}_{last}=1$ |
| Without a predominant provider |  |  |
| Over-dispersion PNC services | Each provider provides only one PNC service | N =M |
| Other multiple providers | Provides the most PNC services among all PNC providers, but do not provide the first or the last PNC service | $n_{i}= max(n_{i})$,  count $max\left( n_{i} \right)>1$,  and $P{NC}_{1st}=0 and P{NC}_{last}=0$ |

Note: Samples were limited to those with at least two prenatal care visits.

PNC: prenatal care. N: total number of visit. $n_{i}$: number of visits to *i*th different provider, i = 1, 2, …..M. M: number of potentially available providers.

**Methods on constructing indices of continuity of care**

COC and predominant PNC providers would then be estimated with the available PNC information among those with at least two PNC visits.^1,2^ A composite index integrated four COC domains: density, dispersion, sequence, and concentration. The composite index was defined as:

$${COMP}_{i}=({UPC}_{i}+{MMCI}_{i}+{SECON}_{i}+{HI}_{i})/4$$

where ${COMP}_{i}$ was the integrated value of continuity of prenatal care for the *i*th observation, UPC (Usual provider of care) measured PNC density, MMCI (Modified modified continuity Index) measured PNC dispersion, SECON (Sequential Continuity of Care Index) measured PNC sequence, and HI (Herfindahl Index) measured PNC concentration. All COC indices range between 0 and 1, with a higher value indicating a higher COC. Formulae can be found in Supplemental Table 4.

Differences in the COC indices were examined using the *student t*-test between pregnancies with a predominant PNC provider and those without.

**Results of the association between COC indices and assigned predominant provider status**

COC indices and predominant providers were estimated for those with at least two PNC visits (89,030 episodes). The index means (standard deviation) were 0.71 (0.20), 0.81 (0.16), 0.63 (0.27), 0.61 (0.23), and 0.69 (0.20) for UPC, MMCI, SECON, HI, and COMP, respectively (Supplemental Figure 1). Supplemental Figure 1 presents the comparison of COC indices by provider category. Pregnancies without a predominant provider were set as the reference group. Other groups with a predominant provider were compared to them. COC indices of pregnancies with a predominant provider were significantly higher than those without (p < .0001), except UPC for the multi-plural group (0.39 vs. 0.40, p = .12).

While this study establishes the methodological foundation for constructing COC indices, the detailed application of our algorithm to generate these indices appears in the supplemental materials rather than the main text. These COC indices serve as the essential basis for predominant provider identification. Our analysis revealed strong alignment between provider categories and COC index scores: pregnancies with exclusive providers demonstrated perfect continuity (COC = 1.0), while those without a predominant provider consistently showed the poorest continuity scores across most indices. This graded relationship validates both our provider classification system and the underlying continuity measurement approach. (Supplemental Figure 1)

1: Jee, S. H., & Cabana, M. D. (2006). Indices for continuity of care: a systematic review of the literature. Medical Care Research and Review, 63(2), 158-188.

2: Saultz, J. W. (2003). Defining and measuring interpersonal continuity of care. The Annals of Family Medicine, 1(3), 134-143.

Supplemental Table 5. formulae for indices of continuity of care.

| Index/Dimension | Formulas |
| --- | --- |
| Density | $UPC=\frac{\bar{N}}{N}=\frac{max(n_{i})}{N}$ |
| Dispersion | $MMCI=\frac{N+0.1-M}{N+0.1-1}$ |
| Sequence | $SECON=\frac{\sum_{i=1}^{N-1} S_{i}}{N-1}$ |
| Concentration | $HI=\sum P_{i}^{2}=\sum_{i=1}^{M} ({\frac{n_{i}}{N})}^{2}$ |
| Composite | ${COMP}_{i}=({UPC}_{i}+{MMCI}_{i}+{SECON}_{i}+{HI}_{i})/4$ |

Notes:

N: total number of visit. $n_{i}$: number of visits to *i*th different provider, i = 1, 2, …..M. M: number of potentially available providers.

Supplemental Table 6. Characteristics of study participants.

| Measures | Count / Mean | Percentage / Standard deviation |
| --- | --- | --- |
| N | 92,836 | 100.0 |
| Age | 26 | 5.6 |
| Race |  |  |
| Whites | 28,432 | 30.6 |
| Blacks | 34,051 | 36.7 |
| Others / Unknown | 30,086 | 32.4 |
| Missing | 267 | 0.3 |
| Severe maternal morbidity | 1,003 | 1.1 |
| Maternal comorbidity score | 16.8 | 17.3 |
| Group consultation | 2,607 | 2.8 |

Note

Severe maternal morbidity: Severe Maternal Morbidity surveillance system: <https://www.cdc.gov/maternal-infant-health/php/severe-maternal-morbidity/icd.html>.

Maternal comorbidity score: transfusion edition, adopted from Leonard 2020: Leonard, S. A., Kennedy, C. J., Carmichael, S. L., Lyell, D. J., & Main, E. K. (2020). An expanded obstetric comorbidity scoring system for predicting severe maternal morbidity. Obstetrics & Gynecology, 136(3), 440-449.

Group consultation: identified with CPT/HCPCS codes, including group encounters for behavioral/mental conditions (90849, 90853, 96153, 96164, 96195, 97154, 97157, 97158, 0366T, 0367T, 0371T, 0372T), group therapy (97150), group nutrition therapy (97804), training for patient self-management (98961, 98962), group educational services (99078), and group obstetric consultation (S9436- S9439, S9442, S9443).


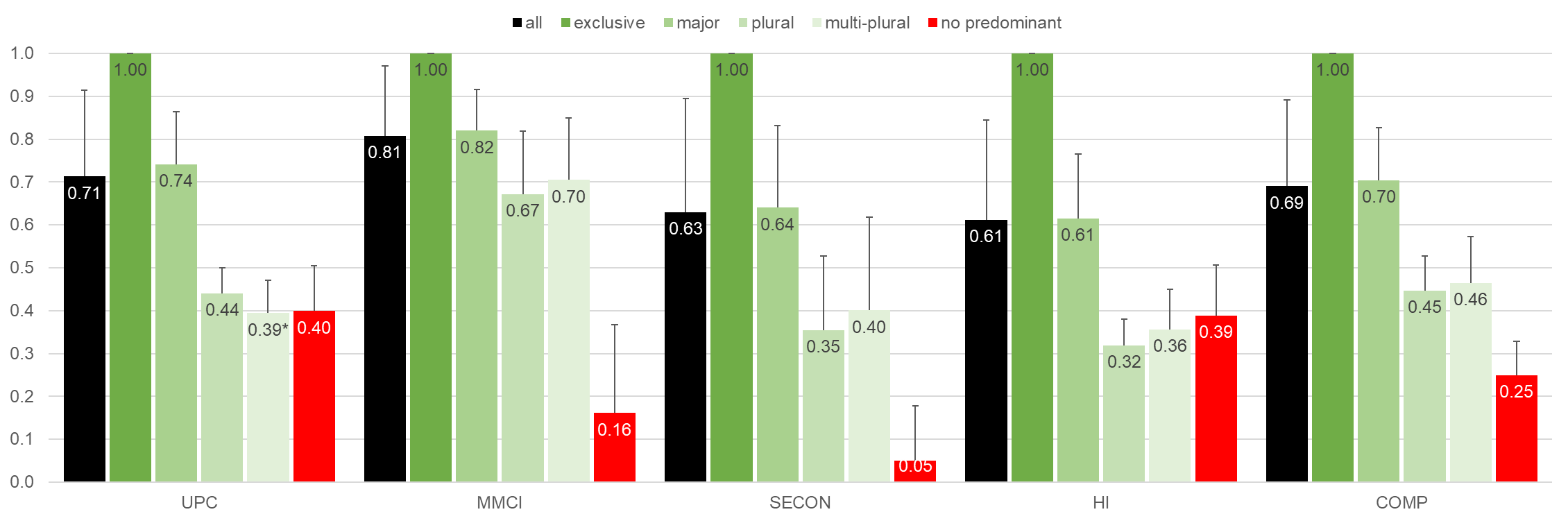


Supplemental Figure 2. Means and standard deviation of Continuity of care for prenatal care by provider types.

Note: UPC stands for Usual provider of care and measures PNC density, MMCI stands for Modified modified continuity Index and measures PNC dispersion, SECON stands for Sequential Continuity of Care Index and measures PNC sequence, HI stands for Herfindahl Index and measures PNC concentration, and COMP stands or composite index and is the arithmetic average of UPC, MMCI, SECON, and HI.

Samples were limited to those with at least two prenatal care visits. Pregnancies would be classified into five groups: with an exclusive, a major, a plural, or a multiple-plural predominant provider, and without a predominant provider.

The non-predominant provider group was set as the reference. All comparisons were significantly different with a p value < .0001, except UPC for the multi-plural group (* p = .12).
